# Evaluation of Eye Gaze Dynamics During Physician-Patient-Computer Interaction in Federally Qualified Health Centers: Systematic Analysis

**DOI:** 10.2196/46120

**Published:** 2023-09-08

**Authors:** Amal Almansour, Enid Montague, Jacob Furst, Daniela Raicu

**Affiliations:** 1 Jarvis College of Computing and Digital Media DePaul University Chicago, IL United States; 2 Department of Mechanical & Industrial Engineering University of Toronto Toronto, ON Canada

**Keywords:** patient-physician-computer interaction, nonverbal communication, Federally Qualified Health Centers, primary care encounter

## Abstract

**Background:**

Understanding the communication between physicians and patients can identify areas where they can improve and build stronger relationships. This led to better patient outcomes including increased engagement, enhanced adherence to treatment plan, and a boost in trust.

**Objective:**

This study investigates eye gaze directions of physicians, patients, and computers in naturalistic medical encounters at Federally Qualified Health Centers to understand communication patterns given different patients’ diverse backgrounds. The aim is to support the building and designing of health information technologies, which will facilitate the improvement of patient outcomes.

**Methods:**

Data were obtained from 77 videotaped medical encounters in 2014 from 3 Federally Qualified Health Centers in Chicago, Illinois, that included 11 physicians and 77 patients. Self-reported surveys were collected from physicians and patients. A systematic analysis approach was used to thoroughly examine and analyze the data. The dynamics of eye gazes during interactions between physicians, patients, and computers were evaluated using the lag sequential analysis method. The objective of the study was to identify significant behavior patterns from the 6 predefined patterns initiated by both physicians and patients. The association between eye gaze patterns was examined using the Pearson chi-square test and the Yule Q test.

**Results:**

The results of the lag sequential method showed that 3 out of 6 doctor-initiated gaze patterns were followed by patient-response gaze patterns. Moreover, 4 out of 6 patient-initiated patterns were significantly followed by doctor-response gaze patterns. Unlike the findings in previous studies, doctor-initiated eye gaze behavior patterns were not leading patients’ eye gaze. Moreover, patient-initiated eye gaze behavior patterns were significant in certain circumstances, particularly when interacting with physicians.

**Conclusions:**

This study examined several physician-patient-computer interaction patterns in naturalistic settings using lag sequential analysis. The data indicated a significant influence of the patients’ gazes on physicians. The findings revealed that physicians demonstrated a higher tendency to engage with patients by reciprocating the patient’s eye gaze when the patient looked at them. However, the reverse pattern was not observed, suggesting a lack of reciprocal gaze from patients toward physicians and a tendency to not direct their gaze toward a specific object. Furthermore, patients exhibited a preference for the computer when physicians directed their eye gaze toward it.

## Introduction

Physicians’ use of computers during consultations may play a role in effective interaction, that contributes to patient satisfaction, adherence to medical care, and trust in physicians [1-5], by increasing information sharing between physicians and patients and developing a clear understanding of conditions and treatment plans [5-8]. Notwithstanding the optimistic results of incorporating technology in clinical settings, other studies have shown the negative side of using technology in encounters. Physicians’ interactions with the electronic health record (EHR) may result in an increased emphasis on the screen (ie, entering or searching patient’s information) than on the patient. This may lead to neglecting the patient in the room and impede effective communication [6,9-12].

This study evaluated a single nonverbal behavior, eye gaze, to provide an overall understanding of the dynamics within physician-patient-computer interaction inside 3 Federally Qualified Health Centers (FQHCs) in Chicago, Illinois. FQHCs provide primary care services to diverse populations, including medically underserved, homeless, and migrant individuals, encompassing various racial and ethnic backgrounds [13,14]. Additionally, FQHCs play a crucial role in mitigating health disparities by providing care to low-income, public insured, and uninsured patients within their local community [15-17]. Therefore, racial and ethnic patients require additional attention due to their lower likelihood of establishing rapport with physicians, receiving empathy from physicians, and being encouraged to participate in discussions during the clinical encounter [18-20].

Eye gaze becomes particularly crucial in situations where speakers and listeners speak different languages. In such cases, listeners rely on the speakers’ eye gaze to enhance their understanding during the interaction [21]. One study focused on conversation patterns and physician gaze shifts between patients and computer screens and evaluated patients’ responses when the physician gaze shifted toward the computer [22]. The study found that physicians are primarily responsible for directing the encounters using gaze and other nonverbal behaviors because they are in charge of computers [22]. Moreover, a study assessed different interactions with physicians and computers, including gazing at the EHR, and their effect on patients’ participation during the encounters [23]. The results showed that the patient was less active the more the physician focused on the computer. At the same time, physicians were in charge of the consultation flow by trying to involve the patients in the conversation while working on computers [23]. Furthermore, another study explored patients’ opinions regarding physicians’ interaction with the EHR by involving patients in watching videos depicting EHR-related activities and asking them about their thoughts on the matter [24]. The study found that most patients preferred physicians who talk and look at them while typing. Additionally, a study evaluated the effect of physicians’ gaze on patients with social anxiety [25]. The study highlighted that patients felt uncomfortable with physicians’ prolonged gaze, leading to diminished trust, emphasizing the need for future research to investigate bidirectional face gaze and its impact on physician-patient dynamics and outcomes [25]. Patel et al [26] explored best practices for integrating technologies into examination rooms. The study provided 12 recommendations aligned with what has been discussed in the literature. In their analysis [26], they found that computers, in addition to maintaining eye contact with the patient, could be used to facilitate patient-centered communication and have a positive effect on the physician-patient relationship.

In previous work, eye gaze patterns were studied dynamically using lag sequential analysis in paper-based [27] and computer-based [28] primary care settings. In the paper-based study [27], there was no prior relationship between patients and physicians and there was no technology presence in the clinic room. In the computer-based study [28], patients were recurring patients, there was a prior relationship between patients and physicians, and physician-patient eye gaze patterns were evaluated in computerized settings, where computers are used. This study represents the naturalistic medical settings with patients from marginalized groups. Patients were new or recurring patients, and the physicians were using computers during the encounters. In contrast to previous studies [27,28] the clinical context in this study included communication patterns specifically with medically underserved patients from different backgrounds, adding a unique perspective to the existing literature. In our previous study using the same data from FQHCs [29], we investigated the consistency of eye gaze patterns between physicians when they look at their patients with the presence of a computer in the encounters using k-means and dynamic time warping. We found common physicians’ eye gaze characteristics between the visits that would be beneficial in designing health technologies. At the same time, the majority of physicians’ gaze patterns showed different behaviors within the same physicians’ visits and between other physicians. Nevertheless, the study lacked patients’ behavior patterns analysis and the behavior patterns evaluation toward the computer.

To improve physician-patient interactions, a perception of EHR’s role in naturalistic settings is required in these clinics that serve the underserved population. The primary research questions for this study are as follows:

1. How is the doctor’s gaze related to the patient’s gaze in computer-mediated health encounters in clinics serving medically underserved patients?
  Do patients follow where the physician gazed?
2. How is the patient’s gaze related to the doctor’s gaze in computer-mediated health encounters in clinics serving medically underserved patients?
  Do physicians follow where the patient gazed?

In approaching these questions, we hypothesize that patients will more frequently follow the gaze of physicians. This is based on the results from previous studies [27,28] that physicians’ eye gaze leads patients’ eye gaze.

## Methods

### Data Set

This study involved a systematic analysis conducted at 3 FQHCs in Chicago in 2014. Although the data set may not be recent, it remains valid for examining nonverbal behavior between physicians and patients in the presence of computers during clinical encounters [29]. During these interactions, physicians used portable computers (laptops). The entire visit was recorded on video and later analyzed by a human coder to identify eye gaze patterns. To capture physicians’ and patients’ eye gaze, 3 cameras were used in the study: a physician-centered camera which was positioned in front of the physician, a patient-centered camera which was placed in front of where the patients are usually sitting, next to the doctor, and a wide-frame camera where you could see a wide view of the room. The original study consisted of 83 visits. However, out of these 83 visits, only 77 included both the physician’s and patient’s faces, making them suitable for eye gaze analysis. The total duration of these visits amounted to 16 hours and 16 minutes. Unfortunately, in the remaining 6 visits, inadequate camera setup in the room resulted in an inability to capture the necessary elements for analysis.

### Ethical Considerations

Patients who participated in the study verbally agreed to take part in the study before and during the recording. Institutional review board approval was obtained from the DePaul University Institutional Review Board (reference number: EM062818CDM-R6) and the study complied with HIPAA (Health Insurance Portability and Accountability Act) regulations.

### Demographics

Demographic characteristics were collected through surveys from doctors and patients. The study involved the participation of 6 female physicians and 5 male physicians. The majority of physicians were of White or Caucasian ethnicity, although there were also physicians from other racial backgrounds, including Asian American or Pacific Islander, and various other racial backgrounds. Patients were coming to the visits for multiple health purposes. All participating physicians were fluent in both English and Spanish. Patient-reported demographics are represented in Table 1. The relationship between patients and their physicians ranges from their first visit to 10 years. First-time patients represent 22 (29%) of all patients. Subjects (patient and physician) participating in the study speak English or Spanish during the visit, 49 (64%) in Spanish, and 27 (35%) in English. A translator was recruited for 1 (1%) patient, who was neither an English nor a Spanish speaker to facilitate the communication between this patient and the physician during the visit.

**Table 1:** Patient demographics data (N=77).

Characteristic		Value
Age (21-70 years), mean (SD)		45.97 (10.92)
**Gender, n (%)**		
	Women	53 (69)
	Men	23 (30)
	Undetermined	1 (1)
**Race, n (%)**		
	Undetermined	24 (31)
	Not indicated	14 (18)
	Mexican	13 (17)
	Black or African American	8 (10)
	Puerto Rican	3 (4)
	Asian	2 (3)
	Caucasian	2 (3)
	Honduran	2 (3)
	Ecuadorian	2 (3)
	Multiracial	2 (3)
	Nicaraguan	1 (1)
	Columbian	1 (1)
	Guatemalan	1 (1)
	Alaskan Native	1 (1)
	Hispanic or Latino	1 (1)

### Coding Scheme

A human coder recorded the start and stop time of the eye gaze behavior in the video. For example, “doctor-gaze-patient” was coded when the doctor looked at the patient. The waiting time, when the patient was in the room waiting for the physician and after the encounter was finished, the physical examination and the time when the gaze was unavailable with either the physician or patient were excluded from the analysis. A coding scheme for eye gaze behavior was adapted from a previous study [28] and adjusted in this study to focus on eye gaze behaviors. It included subjects (patient and doctor), behavior (gaze), and modifiers (patient, doctor, technology, chart, other artifacts, and unknown) for events in each video. The coding process was performed using an open source software, Behavioral Observation Research Interactive Software (BORIS) [30]. The behaviors of the same subject (doctor or patient) were considered mutually exclusive. In the coding scheme, “technology” was used to refer to the portable computers the physicians were using during the encounters which mainly represent the EHR. “Chart” was used to denote charts in the examination room, paper documents with information, or notes written by the clinician during the encounter. “Other artifacts” were the objects or other devices in the room, including phones or tablets, and medicines. “unknown” was used to refer to situations when the subject’s eye gaze was not looking at a particular object while talking and thinking. Since the main focus was only on the physician and patient, looking at another person in the room (ie, family member) was coded as “unknown.” Behavior patterns were identified for doctor-initiated patterns and patient-initiated patterns based on the research questions. Each group had 6 sequential behavior patterns. These included doctor-initiated patterns followed by patient-response behavior patterns and patient-initiated behavior patterns followed by doctor-response behavior patterns (Textbox 1).

**Doctor behavior patterns and patient behavior patterns with the corresponding response behaviors.**

**Initiated behaviors**

Doctor-initiated behaviors:

- Doctor gaze patient (DGP)
- Doctor gaze chart (DGC)
- Doctor gaze other artifact (DGO)
- Doctor gaze technology (DGT)
- DGT
- Doctor gaze unknown (DGU)

Patient-initiated behaviors:

- Patient gaze doctor (PGD)
- Patient gaze chart (PGC)
- Patient gaze other artifact (PGO)
- Patient gaze technology (PGT)
- PGT
- Patient gaze unknown (PGU)

**Response behaviors**

Patient-response behavior:

- PGD
- PGC
- PGO
- PGT
- PGD
- PGU

Doctor-response behavior:

- DGP
- DGC
- DGO
- DGT
- DGP
- DGU

### Analysis

We analyzed the frequency of transitions from each initiated behavior to the next response for all 77 visits, for example, from doctor-gaze-patient to patient-gaze-doctor and vice versa (Table 2 and Table 3). We calculated the percentage of eye gaze per visit. For doctor-initiated behavior, the estimation of eye gaze parameters out of approximately 16 hours of total visits are as follows: doctor gaze chart 0.74 (DGC; 4.6%) hours; doctor gaze other artifact (DGO, 0.22, 1.4% hours); doctor gaze patient (DGP; 6.4, 39.5% hours); doctor gaze technology (DGT; 7.4, 40.5% hours); doctor gaze unknown (DGU; 2.3, 14% hours). For patient-initiated behavior, the estimation of eye gaze parameters in all the visits are as follows, patient gaze chart (PGC; 0.72, 4.2% hours); patient gaze doctor (PGD; 9.1, 53.4 hours); patient gaze other artifact (PGO; 0.4, 2.1% hours); patient gaze technology (PGT; 0.3, 1.7% hours); patient gaze unknown (PGU; 6.2, 38.6% hours).

**Table 2:** Doctor behavior pattern frequencies and the standardized (adjusted) residuals.

	PGO^a^	PGC^b^	PGD^c^	PGT^d^	PGU^e^
DGO^f^	47 (23.69)	2 (–1.91)	57 (0.81)	0 (–1.65)	24 (–7.19)
DGC^g^	5 (–1.53)	107 (22.07)	130 (–1.24)	4 (–1.19)	103 (–7.76)
DGP^h^	37 (–4.68)	86 (–4.72)	1006 (2.05)	22 (–5.04)	1246 (3.02)
DGT^i^	52 (0.04)	71 (–3.56)	706 (–3.54)	84 (9.15)	987 (2.47)
DGU^j^	13 (–2.47)	29 (–2.78)	386 (2.31)	4 (–3.58)	447 (0.78)

^a^PGO: patient gaze other artifact.

^b^PGC: patient gaze chart.

^c^PGD: patient gaze doctor.

^d^PGT: patient gaze technology.

^e^PGU: patient gaze unknown.

^f^DGO: doctor gaze other artifact.

^g^DGC: doctor gaze chart.

^h^DGP: doctor gaze patient.

^i^DGT: doctor gaze technology.

^j^DGU: doctor gaze unknown.

**Table 3:** Patient behavior pattern frequencies and the standardized (adjusted) residuals.

	DGO^a^	DGC^b^	DGP^c^	DGT^d^	DGU^e^
PGO^f^	50 (23.87)	8 (–0.46)	45 (–3.5)	32 (–1.51)	13 (–3.32)
PGC^g^	3 (–1.78)	84 (16.19)	113 (–2.06)	40 (–5.27)	52 (–0.73)
PGD^h^	53 (–4.48)	150 (–4.43)	1417 (4.06)	818 (0.18)	575 (–0.75)
PGT^i^	6 (2.54)	7 (0.73)	42 (0.91)	25 (0.49)	5 (–3.19)
PGU^j^	38 (–3.09)	108 (–2.87)	898 (–2.32)	613 (2.6)	454 (3.01)

^a^DGO: doctor gaze other artifact.

^b^DGC: doctor gaze chart.

^c^DGP: doctor gaze patient.

^d^DGT: doctor gaze technology.

^e^DGU: doctor gaze unknown.

^f^PGO: patient gaze other artifact.

^g^PGC: patient gaze chart.

^h^PGD: patient gaze doctor.

^i^PGT: patient gaze technology.

^j^PGU: patient gaze unknown.

Lag sequential analysis was performed using the Noldus Observer XT 14, a behavioral coding software (Noldus, Wageningen) [31]. After obtaining the frequency of each behavior, we performed Pearson chi-square test for independence to assess the relationships between the variables at *P*=.01. The hypothesis of the test is as follows:

- Null hypothesis: There is no evidence of association between the initiated behavior patterns and the response behavior patterns. For instance, if a doctor gazes at a patient, the patient does not necessarily gaze back at the doctor.
- Alternative hypothesis: There is evidence of association between the initiated behavior patterns and the response behavior patterns. For instance, if a doctor gazed at a patient, patient would gaze back at the doctor.

After that, adjusted residuals were calculated for each table cell. We assumed that adjusted residuals follow a normal distribution. We set a critical value *z*=2.58 and *P*=.01 to indicate a significant association between the initial behavior and the response behavior for both doctor and patient (Table 2 and Table 3). Last, the Yule Q test was performed to estimate the strength of the association between behavior pairs for both doctor and patient to a (–1,+1) range (Table 4) [32]. Negative association of the 2 behaviors indicates the response behavior is not likely to happen given the initial behavior. Zero indicates weak association and the occurrence of the 2 behaviors is random. Finally, positive association indicates a relationship between the initial behavior and the response behavior.

**Table 4:** The Yule Q test for doctor-initiated behaviors and patient-initiated behaviors.

Sequential behavior pairs		Yule Q value
**Doctor-initiated behaviors**		
	DGO^a^-PGO^b^	0.93
	DGC^c^-PGC^d^	0.85
	DGP^e^-PGD^f^	0.06
	DGT^g^-PGT^h^	0.70
	DGT-PGD	–0.10
	DGU^i^-PGU^j^	0.03
**Patient-initiated behaviors**		
	PGO-DGO	0.93
	PGC-DGC	0.77
	PGD-DGP	0.11
	PGT-DGT	0.06
	PGT-DGP	0.10
	PGU-DGU	0.10

^a^DGO: doctor gaze other artifact.

^b^PGO: patient gaze other artifact.

^c^DGC: doctor gaze chart.

^d^PGC: patient gaze chart.

^e^DGP: doctor gaze patient.

^f^PGD: patient gaze doctor.

^g^DGT: doctor gaze technology.

^h^PGT: patient gaze technology.

^i^DGU: doctor gaze unknown.

^j^PGU: patient gaze unknown.

## Results

### Overview

We have provided percentages of each behavior pattern examined in the study. The percentages have been calculated as the ratio between the duration of a specific behavior in all the visits and the total duration of all the recorded visits. Several eye gaze patterns from both physicians and patients are significantly associated (Table 2 and Table 3).

### Doctor-Initiated Behaviors

The results from Pearson chi-square test for doctor-initiated behaviors are as follows: *χ*^2^_16_=1168.3 and *P*<.001. In total, 3 out of 6 doctor-initiated gaze patterns were followed by patient-response gaze patterns, DGO-PGO (doctor gaze other artifact-patient gaze other artifact), DGC-PGC (doctor gaze chart-patient gaze chart), and DGT-PGT (doctor gaze technology-patient gaze technology; Table 2). The Yule Q test’s results agreed with chi-square test results (Table 4). Strong positive associations were shown by 3 out of 6 sequential behavior pairs, DGO-PGO=0.93, DGC-PGC=0.85, and DGT-PGT=0.70 (Table 4). The pair DGP-PGD (doctor gaze patient-patient gaze doctor) was not significant here; however, the pair DGP-PGU (DGP-patient gaze unknown) exhibited a significant relationship (Table 2). DGT-PGT showed a significant relationship (Table 2), and Yule Q results reflected high positive associations of 0.70 (Table 4). High percentages of behavior patterns in the visits for physicians were when they were gazing at computers and when they were gazing at patients.

### Patient-Initiated Behaviors

The results for patient-initiated behaviors are as follows: *χ*^2^_16_=872.51 and *P*<.001. In total, 4 out of 6 patient-initiated gaze patterns were also followed by doctor-response gaze patterns significantly, PGO-DGO, PGC-DGC, PGD-DGP, and PGU-DGU (PGU-doctor gaze unknown; Table 3). Yule Q test results showed that 2 out of 6 sequential behavior pairs showed strong positive associations, PGO-DGO=0.93 and PGC-DGC=0.77 (Table 4). Small positive associations were exhibited by 3 sequential behavior pairs, PGD-DGP=0.11, PGT-DGP=0.10, and PGU-DGU=0.10 (Table 4). High percentages of behavior patterns during the visits for patients were when they were gazing at physicians and when they were gazing at the unknown.

## Discussion

### Principal Findings

The results indicated a statistical significance in the dependency of various eye gaze patterns, both in doctor-initiated and patient-initiated patterns. In total, 3 out of 6 of the doctor-initiated behavior patterns were significant. We found that patients tended to reciprocate eye gaze patterns initiated by physicians when they looked at “other artifact,” “chart,” and “computer.” On the other hand, a significant relationship in DGP-PGU sequence pattern was observed. For instance, if a physician gazes at a patient, the patient does not necessarily gaze back at the physician and most likely is not looking at a specific object (unknown). For patient-initiated behavior patterns, 4 out of 6 sequential pairs were significantly followed by doctor-response eye gaze patterns. We discovered that physicians were inclined to respond to patients’ eye gaze when they looked at “physician,” “other artifact,” “chart,” and “unknown.” However, unlike the previous studies [27,28], the analysis showed that PGD-DGP pair exhibited a significant association. When patients initiated eye contact with their physicians, the study found that physicians predominantly responded by reciprocating the gaze back toward the patients. However, the reverse was not as prevalent as in [27,28]. Although physicians spent a large amount of visit time gazing at patients [29], patients were less frequently responding to doctors’ initiated eye gazes. Moreover, the sequential pair PGT-DGT showed a lack of significant association in contrast with the previous study [28]. Similarly, PGD-DGT sequential pair was not significant in this study, unlike the results in [28] which showed some form of positive interactions with the patients.

Physicians allocated approximately 6.4 out of 16 (39.5%) hours of the encounter to gazing at patients and 7.4 out of 16 (40.5%) hours to gazing at technology.

There could be some interpretations for DGP-PGD insignificant pattern given that patients were from different racial or ethnic groups. However, the lack of data on patients’ race or ethnicity makes it difficult to derive a deeper insight into why the DGP-PGD pattern exhibited different behavior than previous studies in non-FQHC settings [27,28]. These studies [27,28] have shown that physicians’ gaze patterns always influence patients’ gaze patterns (ie, if the physician gazed at the patient, the patient would gaze back at the physician). Moreover, DGU-PGU did not exhibit a significant association in this study, and DGU did not show any significant association with other behaviors. A physician most likely was gazing at unknown objects during the visit when there was not much interaction with the computer. Another possible interpretation is that the physician’s eye gaze was moving between the patient or the computer to the unknown objects during the consultation instead of just focusing on the patient the whole time. In this case, further study is needed to consider these sequences, DGU-DGT-DGP and DGU-DGP-DGT. Moreover, DGT-PGT was significant and showed a strong association with patients who tended to follow the doctors’ gaze at the computer [28]. DGT-PGT pair could be a positive indicator of successfully engaging the patients during the conversation with the computer [33]. Multiple studies suggest that computers can help to improve the capture and sharing of information, which can lead to improved patient outcomes [33-35]. However, the DGT-PGD pattern showed a negative relationship meaning when the doctor was gazing at the computer the patient was gazing at something else except the doctor.

In total, 49 out of 77 (64%) visits were conducted in Spanish, and in some of the other remaining visits, the patients were not fluent in English. The pair PGD-DGP shows a good indicator of successfully engaging the patients in the conversation even though the majority of the visits were not conducted in English. Another explanation could be that Spanish is not the first language for most of the participating physicians and that is why they tend to follow patients’ eye gaze [21]. The sequential pair PGT-DGT did not show any significant relationship and had a very negligible association (0.06). Patients were not positioned in front of the computer and were not asked to use the computer during the encounter. Likewise, chi-square analysis for PGT does not show any significant results with any other sequential behaviors. However, physicians can share the screen with the patients by moving the computer toward them to discuss the information or results. In contrast to the findings in this study around doctor-initiated gaze at technology, physicians tended not to follow patients’ gaze at the computer when initiated by the patient. When a patient gazed at the computer, the physician was mostly focusing on other things and that could be indicated from the results. The physician could be reviewing other work (ie, reading a chart or looking at medicine) or looking at the patient. The physicians may also know that the technology in the encounter is not patient-centered and that is why it is not necessary to follow patients’ eye gaze. Furthermore, the sequential pair PGD-DGT was not significant in this study. This pair PGD-DGT could also imply the process of encouraging patients to participate more during the encounter and ask questions. In this scenario, we would expect to see a patient gaze at the doctor, then the doctor gaze back at the patient, and finally, the doctor gazes at the technology to enter or retrieve information. More analysis is needed to include a second lag to test this sequence (PGD-DGP-DGT). However, it was observed that physicians predominantly followed patient-initiated eye gaze patterns, indicating increased engagement in conversations with patients and possibly demonstrating greater empathy toward them [36]. Last, based on the findings, the second most prevalent behavior pattern observed in patients during the visits was characterized by a lack of focus or the absence of directed gaze toward a specific object or target. This pattern accounted for approximately 6.2 hours out of 16 hours (equivalent to 38.6% of the total duration). The pair PGU-DGU showed a significant relationship. When the patient was not looking at a specific object, the physician was also not focusing on a specific thing generally. Therefore, the findings from the PGU-DGU pair support the idea that patients, during encounters with physicians, did not exhibit a specific object of focus. Instead, their gaze tended to wander around the room, suggesting that patients could benefit from clearer guidance on where they should direct their attention.

### Comparison With Prior Work

The time the physician spent gazing at patients and gazing at the computer is consistent with previous study [28], which showed that physicians spent more than one-third of the visit’s length gazing at the computer. For doctor-initiated behavior, the DGT-PGD pair showed a significant negative relationship. Patients tended to gaze at everything else except the physician when the physician was gazing at the computer. Furthermore, the sequence pair DGP-PGD did not show significant associations in this study unlike the findings in previous studies [27,28]. Physicians’ eye gaze behaviors toward their patients could be varied [29]. Nevertheless, the responses from their patients were not significant. However, DGP-PGU behavior showed a significant response. In previous studies, paper-based encounters [27] and technology-based encounters [28], physicians’ eye gaze behaviors lead patients’ eye gaze behaviors all the time during the interaction. Therefore, interventions such as redesigning technologies or training directed at physicians are likely to be successful in influencing patients’ behaviors and the dynamics of the encounter [28]. However, in this study, not all doctor-initiated gaze patterns were followed by patients’ gaze patterns. In other words, patients’ eye gazes were not always following doctors’ eye gazes and most of the time patients’ eye gazes were not focused on specific things (unknown).

### Strengths and Limitations

Our study provides an essential contribution to the literature by shedding light on the experiences of minority groups and underserved populations within the FQHC context. It highlights potential areas where health care providers in such clinics can further optimize their use of EHR systems to improve communication and overall patient care. This study is the largest naturalistic quantified ethnographic study of clinical encounters that primarily serve marginal groups we are aware of. By providing a broader perspective on the directions of eye gaze in underserved clinics, we believe this study sheds light on the nature of patient-physician interactions in these settings and contributes to the design of health information technology.

However, a key limitation of the study is a lack of sufficient data to fully comprehend cultural and language differences, as well as analyze the impact of racial and ethnic concordance between physicians and patients. This limitation restricts the ability to fully understand the underlying causes of these disparities and draw definitive conclusions solely based on the findings of this study. Thus, it is imperative to collect more data and investigate additional questions related to culture and language in order to facilitate more comprehensive analyses in future research.

### Practice Implications

The differences observed between doctor-initiated and patient-initiated gaze patterns in clinics serving medically underserved patients present a potential challenge for technology designs. The influence of patients on physicians’ behaviors suggests that a shift toward patient-centered technologies may be more important. These findings underscore the significance of patients’ roles in medical encounters. Physicians can benefit from patients’ interest in technology by encouraging them to engage with the information displayed on the screen and maintaining patient-centered communication. Additionally, implementing simplified screen designs in EHR systems can facilitate education for diverse patients during visits. Further research in diverse settings is necessary to inform the design of future EHR systems that effectively enhance doctor-patient communication in these clinics.

### Conclusions

This study investigated the bidirectional gaze patterns among physicians, patients, and computers in clinic settings primarily catering to marginalized populations. Our hypothesis was that physicians’ eye gaze would consistently lead to patients’ eye gaze, as observed in previous studies [27,28]. However, we found that not all gaze patterns initiated by physicians were reciprocated by patients. Conversely, physicians’ eye gazes predominantly followed patients’ initiated gazes. Interestingly, the sequence pair DGP-PGD did not show any significant relationship. These findings may provide some form of engagement and show more compassion and empathy with patients [36]. Interestingly, the sequence pair DGP-PGD did not exhibit a significant relationship, while the pair DGP-PGU demonstrated a significant relationship. Patients hesitated to look back at the physicians during the interaction. Additionally, patients showed interest in technology based on DGT-PGT results.

The results also showed that patient-initiated gaze with technology was not significant. This may indicate that computer design in those settings is not targeted at patients, which means that any intervention that influences screen or EHR information sharing will likely need to be encouraged [33,37]. The findings from patient-initiated gaze patterns illustrate the importance of designing patient-centered technology [28]. These findings offer evidence indicating potential differences in communication patterns between patients and physicians in clinics that cater to medically underserved individuals from diverse backgrounds.

## Abbreviations

BORIS: Behavioral Observation Research Interactive Software
DGC: doctor gaze chart
DGO: doctor gaze other artifact
DGP: doctor gaze patient
DGT: doctor gaze technology
DGU: doctor gaze unknown
EHR: electronic health record
FQHC: Federally Qualified Health Center
HIPAA: Health Insurance Portability And Accountability Act
PGC: patient gaze chart
PGD: patient gaze doctor
PGO: patient gaze other artifact
PGT: patient gaze technology
PGU: patient gaze unknown

## Data Availability

The data used in this study are not publicly available due to privacy restrictions and ethical considerations to protect patient privacy and confidentiality. Researchers interested in accessing the data may submit a formal request to the institutional review board and the corresponding author, outlining the purpose and scope of the intended analysis. Requests for data access will be reviewed by the institutional review board to ensure compliance with applicable regulations and guidelines.
